# Advancing Circular Economy Implementation for High-Speed Train Rolling Stocks by the Integration of Digital Twins and Artificial Intelligence

**DOI:** 10.3390/s25206473

**Published:** 2025-10-20

**Authors:** Lalitphat Khongsomchit, Sakdirat Kaewunruen

**Affiliations:** Department of Civil Engineering, University of Birmingham, Birmingham B15 2TT, UK; lxk301@student.bham.ac.uk

**Keywords:** digital twins, machine learning, artificial intelligence, rail, rolling stock, circular economy

## Abstract

This paper presents a state-of-the-art review on the integration of digital twins and artificial intelligence to advance the circular economy and the 10R principles implementation in high-speed train rolling stock. Rolling stock generates substantial waste at the end of its service life, yet the application of the circular economy and the 10R principles (Refuse, Rethink, Reduce, Reuse, Repair, Refurbish, Remanufacture, Repurpose, Recycle, and Recover) in this domain remains limited compared with infrastructure. The review analyses 47 studies retrieved from Web of Science and IEEE Xplore, focusing on digital twin applications in railway infrastructure and rolling stock, and machine learning techniques. Findings reveal that most studies concentrate on data management and efficiency improvement, while only a few explicitly address the circular economy and 10R principles. A comparative analysis of high-waste components against current machine learning applications further highlights critical gaps. To address these, an automated workflow is proposed, incorporating digital twins, artificial intelligence, and the 10R principles to support condition monitoring and sustainable resource management. The study provides insights and research directions to enhance sustainability in railway asset management.

## 1. Introduction

**Figure 1:**
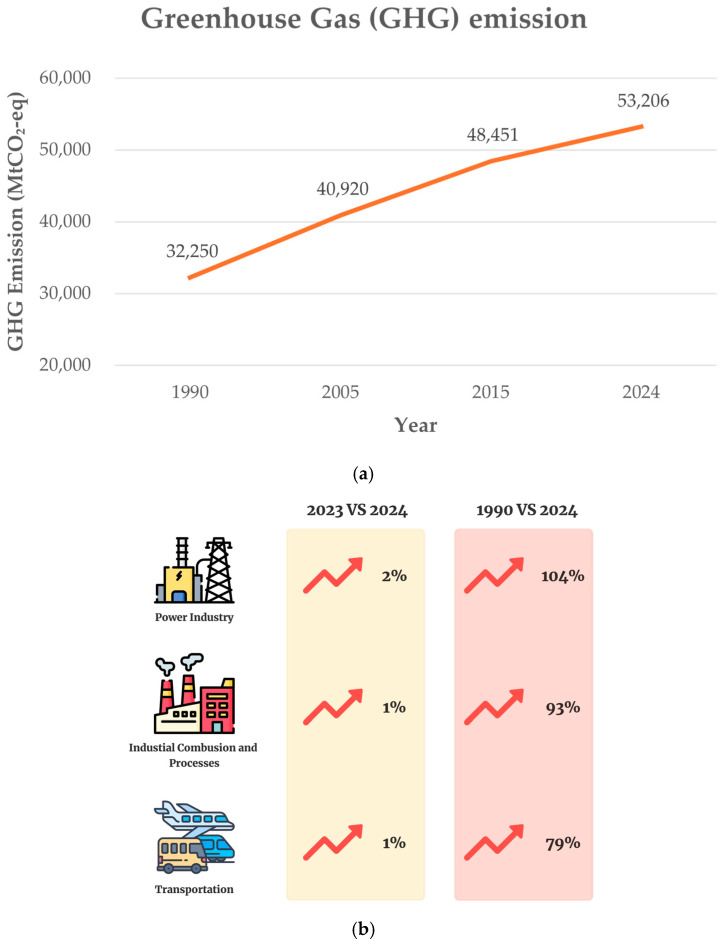
Global greenhouse gas (GHG) emission trends [2]. (**a**) The trend of global GHG emissions from 1990 to 2024 shows a significant increase of nearly 20,000 Mt CO_2_eq over the past 34 years. (**b**) Comparison of GHG emission growth rates among the three highest-emitting sectors, power industry, industrial combustion and processes, and transportation, between 1990–2024 and 2023–2024.

Climate changes refer to long-term changes in temperatures and weather patterns. Since the 1800s, human activities have been the main cause of climate change, mostly because of burning fossil fuels. Fossil fuels combustion produces greenhouse gas emissions. The main greenhouse gases responsible for climate change are carbon dioxide and methane. Carbon dioxide and methane come from using gasoline for driving a car or burning coal for heating a building. Agriculture, oil, and gas operations are major sources of methane emissions. Energy, industry, transport, buildings, agriculture, and land use are among the main sectors that cause greenhouse gases Nations [1]. According to the European Commission’s 2025 report on GHG Emissions of all countries, global GHG emissions have shown a substantial upward trend since 1990. Over the past 34 years, total GHG have increased by nearly 20,000 Mt CO_2_-equivalent, reaching over 50,000 Mt CO_2_-equivalent in 2024. Among all sectors, the power industry, industrial combustion and processes, and transportation have been identified as the top three contributors to this increase, together accounting for the largest share of global GHG growth between 1990 and 2024 [2], as shown in Figure 1.

**Figure 2:**
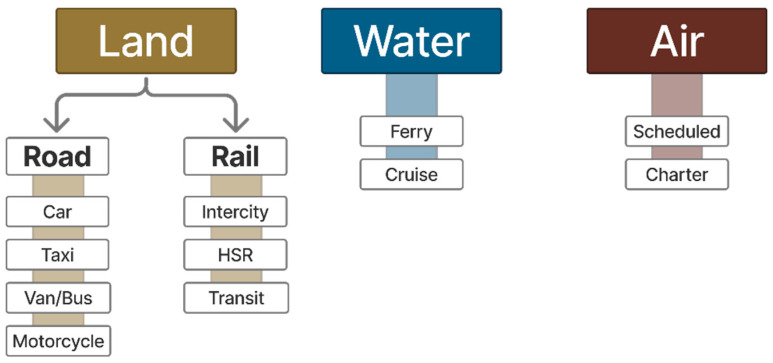
Modes of transport, adapted from Rodrigue [3].

**Figure 3:**
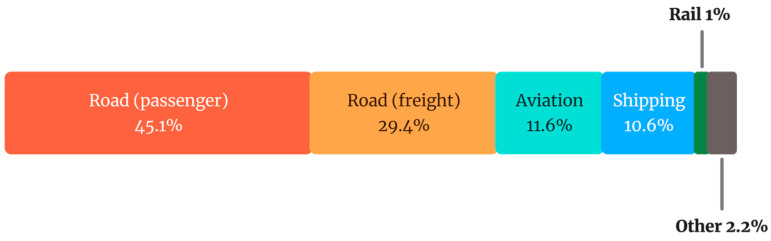
Share of CO_2_ emissions by transportation mode (adapted from Ritchie [4]).

As mentioned, human activities, particularly transportation, are part of the cause of climate change. To comprehend its environmental impact, it is important to identify the modes of transport, generally categorised as land, water, and air. Each mode is characterised by technical, operational, and commercial attributes. Technical characteristics refer to features such as speed, capacity, and technology, while operational characteristics relate to the context in which modes operate, including speed limit, safety conditions, and operating hours [3]. An overview of the modes of transport is provided in Figure 2, and a comparison of CO_2_ emissions, one of the key greenhouse gases, among different transportation modes is provided in Figure 3 [4].

In terms of greenhouse gas emissions, automobiles contribute 20%, whereas railways, despite carrying 11% of goods across European countries, only contribute less than 0.6% of emissions [5]. Although this appears small compared to automobiles, according to data from the European Environment Agency, CO_2_ emissions from the railway industry in the EU-27 are estimated at approximately 3.3 million tonnes, a significant consideration for greenhouse gas contributions from the transportation sector [6]. Globally, rail transport is widely used, with the total passenger travel distance amounting to approximately 2.47 trillion passenger-kilometres (Pkm) in 2022 [7]. Asia, which includes several developed and developing countries such as China, Japan, Thailand, Laos, and other Southeast Asian nations, contains the largest part at 1.79 trillion (Pkm), driven by the need to increase capacity and reduce traffic congestion. Furthermore, Thailand is developing the high-speed train system to create a connectivity route with other nations, both locally and globally, in accordance with the Belt and Road Initiative (BRI) supported by the Government of the People’s Republic of China [8]. While the high-speed railway system developments offer substantial benefits for mobility and economic growth, environmental concerns are also raised regarding the management of high-speed train rolling stock.

Rolling stock’s improper end-of-life management results in significant waste generation, generally more than other vehicle types. Only 75% of the end-of-life rolling stocks were made of recyclable metals. It means that the rest of the end-of-life rolling stock will be wasted in a landfill [9]. Since train rolling stock is a resource-intensive system, a large quantity of materials is required for its manufacturing and assembly. The total mass of freight train rolling stock can reach approximately 8,000,000 kg, passenger train (Electric Diesel) is 168,373.5 kg, and high-speed train rolling stock is 265,000 kg [10]. Most of the mass comes from steel and aluminium alloys, which are among the most resource-demanding but recyclable materials used in transport manufacturing. These figures illustrate the substantial material input associated with rolling stock production and highlight the importance of implementing sustainable resource management strategies in the railway industry. In the process of ore smelting, the current challenge does not stem from insufficient availability of resources but rather from the declining quality of ores. Lower ore grades require greater extraction and processing, which increases the consumption of energy, water, and land. This intensifies the environmental burden, leading to higher GHG emissions [11].

**Figure 4:**
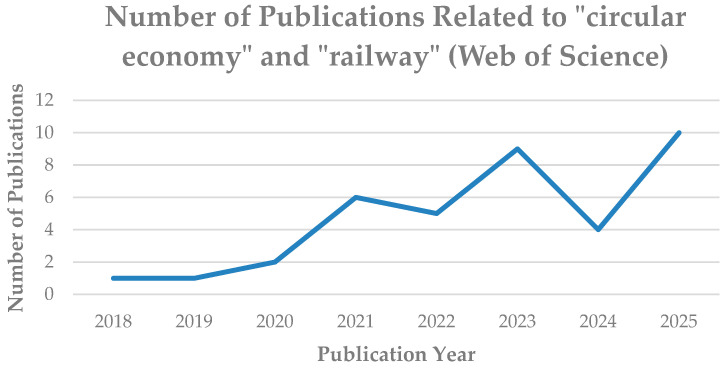
Publications trend on “circular economy” and “railway” (Web of Science, 2018–2025).

Consequently, many initiatives seeking not only to lower landfill use but also to reduce carbon emissions during vehicle operations have started to support the recycling and reuse of vehicle waste. This approach aligns with the concepts of the circular economy. The circular economy plays a crucial role in reducing greenhouse gas emissions by reducing waste and maximizing material reuse. Circular economy helps to reduce demand for new resources by extending the lifespan of materials in rolling stock, therefore supporting environmental sustainability [12]. Moreover, the circular economy is applied through the reuse of in-situ materials in the subgrade, achieving approximately 30% reduction in carbon emissions [13], while in the sub-ballast layer, the integration of reclaimed asphalt pavement and end-of-life tire rubber contributes to a 20–40% reduction in global warming [14]. To highlight the importance of applying the circular economy in the railway industry, the amount of research implementing the circular economy concept in the railway sector has been increasing, as illustrated in Figure 4.

In today’s world, where digitalisation is increasingly used and the Fourth Industrial Revolution is coming, the implementation of tools such as digital twins has gained growing attention. Digital twins refer to virtual representations of physical assets, enabling bidirectional transfer or sharing of data between the physical and virtual models. Digital twins can perform across multiple sectors, such as conducting in-depth analysis of physical twins, simulating health conditions of physical twins, tracking the status throughout the lifecycle, predicting the performance, and facilitating real-time control of the physical twins [15]. However, the adoption of digital twins in high-speed train rolling stock, especially in the area of the circular economy, remains limited. To enable the circulation and utilisation of materials in high-speed train rolling stock and to extend their retention within ‘ material lifecycle in alignment with circular economy principles, this study conducts a state-of-the-art literature review to identify research gaps for future research.

This state-of-the-art review is organised as follows: In Section 2, the circular economy and 10R principles are demonstrated, and the instances of research that have implemented these concepts within the railway sector are discussed. This section also provides an overview of digital twins technology to illustrate the potential of digital twins in enhancing the implementation of the circular economy and 10R principles in the railway sector. Section 3 contains the methodology for selecting relevant studies based on the chosen keywords, including the search strategy, databases used, and inclusion/exclusion criteria for the state-of-the-art review. Section 4 includes the results of applications of digital twins and machine learning in rolling stock and a discussion on how these applications can enhance the implementation of the circular economy in high-speed train rolling stock. This section also proposes an automated workflow to enable circular economy and condition monitoring for the assets. Lastly, Section 5 presents a conclusion and outlines directions for future studies.

## 2. Sustainability in the Railway Industry

### 2.1. Circular Economy and 10R Principles

**Figure 5:**
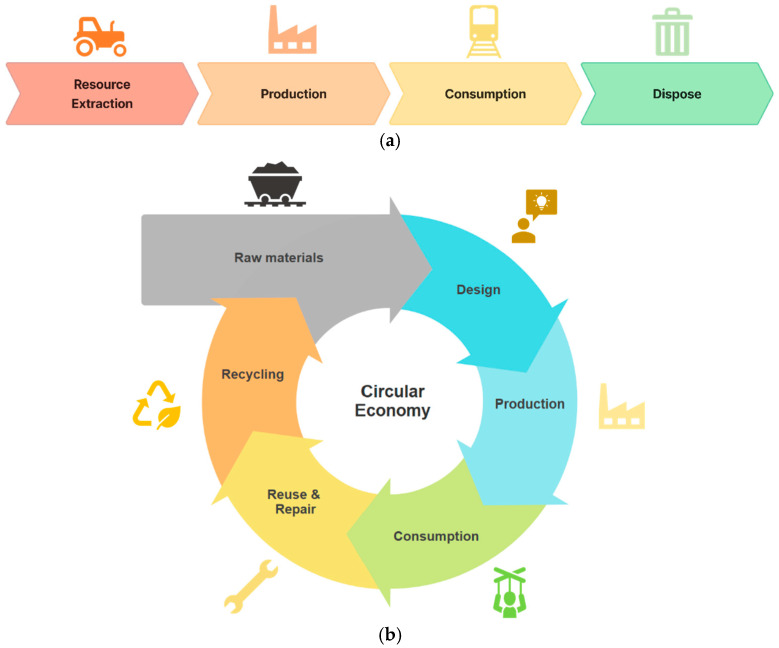
Linear and Circular Economy Models: (**a**) the Linear Economy model, where resources flow in a straight line from extraction to disposal; (**b**) the Circular Economy model, where resources are continuously reused, repaired, and recycled to minimise waste.

The utilisation of raw materials has adhered to a linear economy model, a ‘take-make-dispose’, wherein material is introduced into the production process but are finally discarded at the end-of-life of the finished product, rather than being reused or recycled (as shown in Figure 5a). However, in recent years, the circular economy (as shown in Figure 5b), which advocate reducing raw material consumption, have gained significant attention. Particularly since the introduction of the United Nations’ Sustainable Development Goals (SDGs), the circular economy has been increasingly promoted as a global agenda.

**Figure 6:**
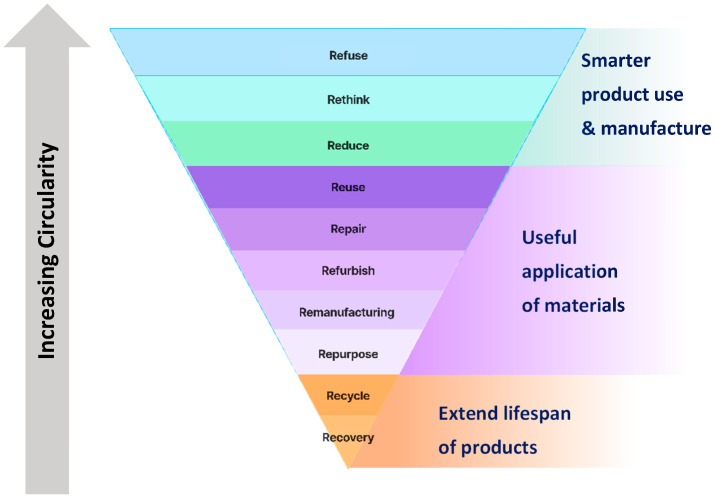
The 10R principles for circular economy [16].

According to José Potting [16] the Policy Report, circularity is categorized into three categories, referred to as the 10Rs, as seen in Figure 6.

To enhance the understanding of the 10R principles, the meaning of each “R” is described, starting from the strategies that exhibit the highest degree of circularity, as follows:

1. Refuse: Make a product redundant by abandoning its function or by offering the same function through a radically different product.
2. Rethink: Make product use more intensive through design or service innovation.
3. Reduce: Increase efficiency in product manufacture or use by consuming fewer natural resources and materials.
4. Reuse: Allow another consumer to reuse a discarded product that is still in good condition and fulfils its original function.
5. Repair: Conduct repair and maintenance of a defective product so it can continue to be used with its original function.
6. Refurbish: Restore an old product and bring it up to date to meet current standards.
7. Remanufacture: Use parts of discarded products in a new product with the same function.
8. Repurpose: Use a discarded product or its parts in a new product with a different function.
9. Recycle: Process materials to obtain the same or lower quality for reuse.
10. Recover: Perform incineration of materials with energy recovery.

In previous studies, the circular economy and the 10R principles have been partially implemented in the railway sector. To illustrate the examples of the application of circular economy in the railway sector, Table 1 presents examples of relevant studies identified through a Web of Science search engine using the keywords “circular economy” and “railway”. The table demonstrates that the implementation of the circular economy and 10R principles in the railway sector is predominantly concentrated on infrastructure rather than rolling stock.

Table 1 illustrates that Circular Economy applications in the railway sector mostly emphasise infrastructure, while research on rolling stock is very scarce. This disparity might be attributed to the structural complexity of rolling stock systems, including multiple components and subsystems. The implementation of a circular economy, such as reuse, remanufacturing, or recycling, is further limited by the variety of materials utilised in manufacturing.

To better understand material-related challenges, the following section provides an overview of the main components and materials that constitute rolling stock systems. The analysis focuses particularly on high-speed train rolling stock, which forms the primary scope of this study.

### 2.2. Material Analysis of High-Speed Train Rolling Stock

This state-of-the-art review, as mentioned in Section 1, concentrates on high-speed train rolling stock. To offer a general overview of rolling stocks, as specified by Silva and Kaewunruen [22], this section briefly presents the types of train rolling stock. Following this, the materials and components of high-speed train rolling stock are detailed.

#### 2.2.1. Types of Train Rolling Stock

This subsection will present the types of rolling stock that are classified according to their functions and services. According to European Practice, rolling stock can be classified into several types. However, in this study, rolling stocks will be divided into 3 types.

- Passenger Trains

Passenger trains, such as a metro, tram, light rail, or urban rail vehicle, are designed to transport passengers and typically consist of elongated carriages meant to operate at higher speeds. It can be a self-propelled railcar or a combination of one or more locomotives and one or more trailers. The primary function of passenger trains is to facilitate passenger transportation between stations. They adhere to fixed schedules and generally accommodate more passengers than other types of trains.

- Freight Trains

Freight trains consist of locomotives and freight wagons or freight cars, facilitating the transportation of materials and goods. Utilizing trains for freight transportation can offer significant economic benefits and efficiency compared to road transport.

- High-speed Trains

High-speed trains fall within the category of long-distance passenger trains. These trains can achieve speeds surpassing 250 km/h and operate on specially designated tracks engineered to accommodate high speeds. Japan’s Shinkansen is the pioneering example of a successful high-speed passenger rail system. In Thailand, the high-speed rail system project connecting Thailand and China, which is officially titled “The Project on Bangkok–Nong Khai HSR Development,” has commenced. The construction in Thailand began with Section 1: Bangkok-Nakhon Ratchasima, covering 250.77 km, which is currently 45.56% complete [8]. As mentioned in Section 1, the development of a high-speed rail system not only enhances mobility and economic growth but also potentially presents environmental impacts arising from the waste generated by high-speed train rolling stock. So, to enable the application of circular economy and 10R principles, the materials and components of high-speed train rolling stock are detailed in the following section.

#### 2.2.2. Material Analysis of High-Speed Train Rolling Stock

Material Analysis of rolling stock components provides insight into their potential for 10R principles, which are key considerations for the circular economy. In the study conducted by Kaewunruen, et al. [10], a summary of the total weight, recyclability rate (%), and recoverability rate (%) of rolling stock was provided in Table 2, comparing three different types of rolling stock.

#### 2.2.3. Circularity Potential of Key Components

**Figure 7:**
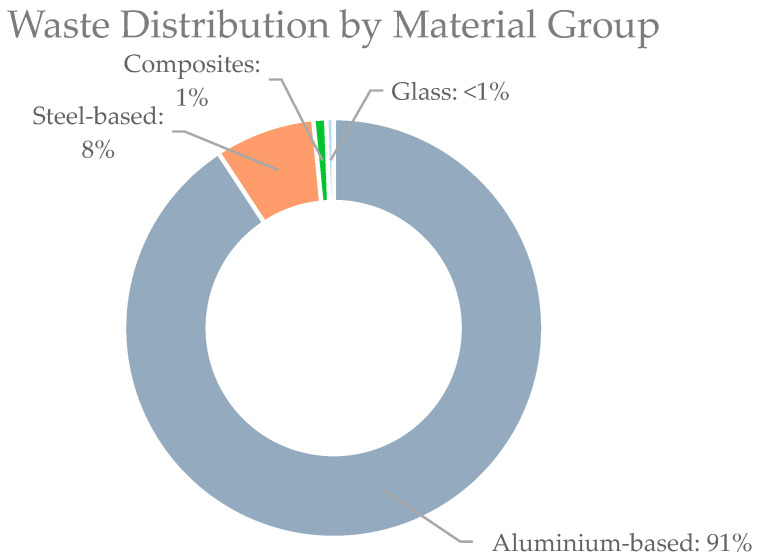
Waste distribution by material group from the top 15 waste-generating components of the high-speed train.

As mentioned in the previous sections, the components of high-speed trains are divided according to the weight of waste generated, ranked from the highest to the lowest in terms of weight (in kilograms). From the 36 components identified in Table 3, the 15 components with the highest waste weight are presented in Table 3, enabling a more practical and effective evaluation of circularity. Following the identification of the top 15 waste-generating components, the waste was categorised by material type into four categories: Aluminium-based, Steel-based, Composites, and Glass, as illustrated in Figure 7.

Following the material classification, a circularity assessment is conducted for each group with specific attention to the 10R principle.

- Aluminium

As shown in Table 3, aluminium-based materials constitute the primary component of waste generated from high-speed train rolling stock. The material possesses a lightweight property that minimizes weight by approximately 10%. Furthermore, the recyclability and recovery rate could exceed 95% [10]. According to Passarini et al. [23] a significant portion of recycled aluminium, up to 75%, in Europe is utilized within the transportation sector.

- Steel

Steel is recognized for its potential to be recycled up to 100% for the production of new steel products. If steel maintains its qualities, it can be recycled. As shown in Table 3, components that are made of steel-based materials include wheels, bogie frame, main transformer, bogie transom, motor suspension coil, and gearbox.

- Glass

Apart from aluminium and steel, glass represents almost 1% of the overall waste. In the context of the circular economy and 10R principles, glass is a reusable material. Used glass is processed into cullet, which is subsequently combined with sand, soda, and limestone before being returned to the manufacturing process [10]. As shown in Table 3, the waste weight of glass amounts to 490.3 kg. Glass can be recycled into various products, such as aggregates in concrete, ceramic sanitary ware, fiberglass insulation products, and recycled glass countertops [10,24].

### 2.3. Digital Twins Technology

**Figure 8:**
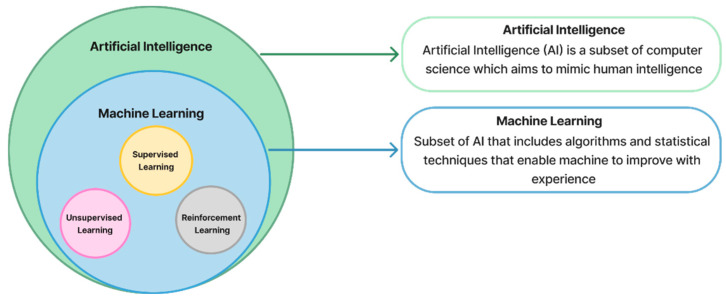
Brief definitions of Artificial Intelligence, machine learning (adopted from Uc Castillo, et al. [28]).

Before the emergence of digital twin technology, Artificial Intelligence (AI), officially introduced during the 1956 Dartmouth Conference, has since evolved significantly, driving advancements in computational power, machine learning, and big data analytics. These advancements have established the foundations for transformational technologies such as digital twins [25,26]. Machine learning and Deep learning, which are considered essential subsets of AI, play a particularly important role in this evolution (as shown in Figure 8).

**Figure 9:**
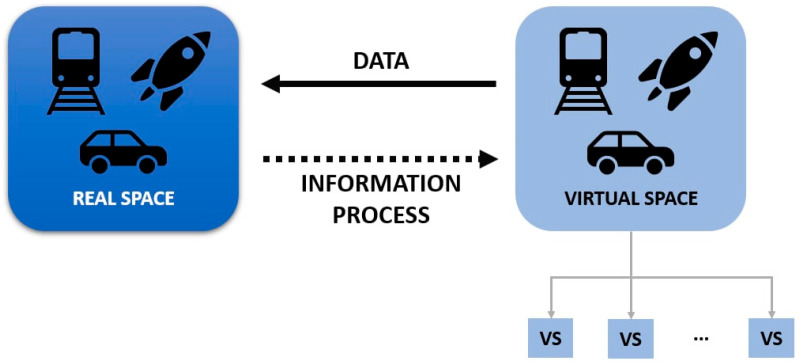
Conceptual ideal for product lifecycle management (PLM) by Grieves and Vickers [27].

The emergence of digital twins has been largely driven by advancements in Artificial Intelligence (AI). Digital twins are a virtual representation of physical systems. This kind of technology does simulations based on real data. The concept of digital twins was informally introduced by Michael Grieves in 2002 during his presentation with the title “Conceptual Ideal for Product Lifecycle Management (PLM)”. Grieves’ DT model, later detailed in publications [26], consist of three key components: (1) a real space containing a physical object; (2) a digital space containing a virtual object; and (3) the link for data flow from real space to virtual space, and information flow from virtual space to real space [27] as shown in Figure 9.

After presenting studies that demonstrate the implementation of circular economy within the railway sector in Section 2.1, Table 4 illustrates how various digital technologies, such as Digital twins and Artificial Intelligence, have been employed across multiple domains to enhance circular economy practices.

**Table 4:** Overview of Digital Technology Applications Supporting the Circular Economy Across Multiple Domains.

Domain	Objective	Digital Technology Involved	Circular/Sustainability Relevance	Reference
Supply Chain Management	To review and synthesise various Industry 4.0 technologies and their environmental sustainability implications within supply chain management.	DTs, AI, IoT, blockchain, cloud computing	Waste reduction, emission control, traceability, reverse logistics and CE	Challouf, et al. [29]
	To map the theoretical, contextual, and methodological evolution of net-zero supply chain management and identify research gaps in digital and circular strategies.	DTs, AI, Data Analytics, Life Cycle Assessment Frameworks	Highlights the integration of circular economy and digital transformation themes to support data-informed decarbonisation and sustainable supply chain transitions.	Raman, et al. [30]
Energy Storage	To review modelling and simulation approaches for optimising electrode drying and predicting defect formation.	DTs/ML, etc.	Green solvents and solvent recovery within drying systems	Mujumdar, et al. [31]
	To explore how digitalisation and digital twins can enable circular and efficient end-of-life management in the lithium-ion battery value chain.	DTs	Battery recycling	Cardenas-Sierra, et al. [32]
Urban system	To propose and validate an integrated SSCC architecture linking digital, governance, and data systems to support circular urban transformations.	DTs, AI, IoT, Drones, Participatory Platforms	Integrated digital and governance frameworks enable circular strategies through waste–energy–information nexus	Velasquez-Mendez, et al. [33]
Construction	To review how digital technologies enhance circularity and life cycle management in the construction sector	DTs, BIM, IoT, Blockchain, Big Data	Optimises resource use, improves traceability, and advances circularity in line with EU sustainability standards	Gondak, et al. [34]
	To develop and validate a predictive digital twin–based system integrating BIM, IoT, and AI for efficient and sustainable construction resource management.	DTs, BIM, IoT, ML	Enhances real-time monitoring and predictive control of site resources, reducing waste and improving efficiency to support circular economy.	Elghaish, et al. [35]
	To examine the role and benefits of digital twinning in facilitating the transition to a circular economy in the construction industry	DTs	Waste reduction, resource optimisation	Awodele, et al. [36]
	To conceptualise and examine the role of digital twins as enablers of circular economy and sustainable development goals in the construction and manufacturing sectors.	DTs	Demonstrates how DT-driven information enhances recyclability, reusability, and sustainability.	Ali, et al. [37]
Port Energy System	To propose a digital twin–based dynamic optimisation framework for zero-carbon port energy systems integrating renewable management and carbon accountability.	DTs, Federated Learning, Hybrid Quantum–Classical Optimisation, Blockchain, Adversarial Reinforcement Learning	Real-time optimisation for renewable utilisation, carbon reduction, and energy efficiency	Li, et al. [38]
Waste Management	To introduce and validate a digital twin–based methodology for optimising organic waste management processes	DTs, Cloud Architecture, IoT-enabled Monitoring	Resource recovery and demomstrating scalable potential for circular economy applications in waste management	Vargas, et al. [39]
Manufacturing	To review engineering innovations and technologies advancing PVC recycling and circular manufacturing integration.	DTs, AI, Robotics, Hyperspectral Imaging	Promotes digitally integrated recycling systems supporting circular manufacturing and sustainable polymer reprocessing.	Chidara, et al. [40]

## 3. Methodology

### 3.1. Research Gap Identification Approach

**Figure 10:**
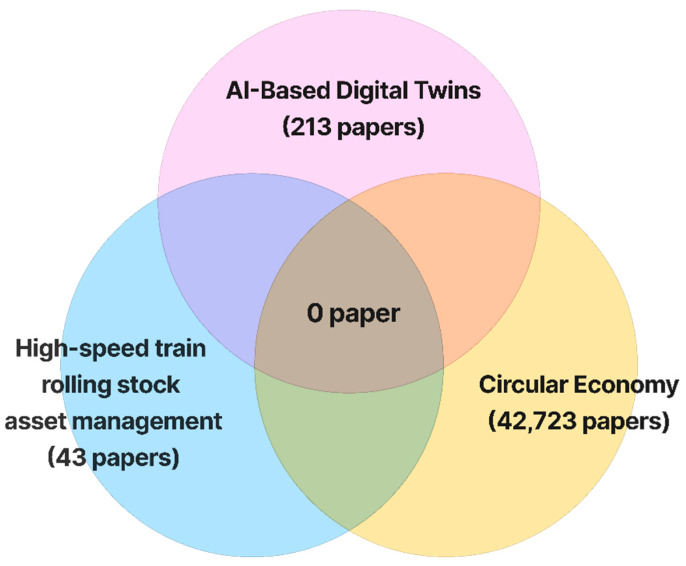
Venn Diagram.

First, to achieve a comprehensive review of the relevant literature, a Venn diagram was created as a visual tool to examine potential gaps between existing research topics, identified through selected keywords, for defining future research directions and their novelty. The keywords used were “AI-based digital twins”, “High-speed Train rolling stock asset management,” and “Circular Economy,” using the Web of Science as a search engine to determine the number of research studies conducted on those fields. The Venn diagram for this study is shown in Figure 10.

From the results of the Venn Diagram, using these three keywords, it is demonstrated that no previous research has integrated AI-based digital twins, High-speed train rolling stock asset management, and circular economy within the same research scope

### 3.2. Paper Screening and Selection

As stated in Section 2.3, machine learning (ML) enables AI and digital twins. Consequently, the publications are selected from both fields: digital twins and machine learning. In the digital twins part, the keywords “Digital twins” AND “Railway” and “Digital twins” AND “Rolling stock” were used to search for studies related to this topic, and in the machine learning domain, the keywords “Machine Learning” AND “Rolling Stock” were used in the screening and selection phase. The review was performed utilising two scholarly databases: Web of Science and IEEE Xplore.

The review process was undertaken in four key steps:

1. Retrieve publications from the Web of Science and IEEE Xplore database using the defined keywords.
2. Eliminate irrelevant publications by screening abstracts, introductions, and conclusions to ensure alignment with the inclusion criteria.
3. Conduct a detailed review of the full content of all relevant publications filtered in step 2.
4. Undertake a comprehensive analysis, categorizing the publications based on applications, attributes, and functionalities.

**Figure 11:**
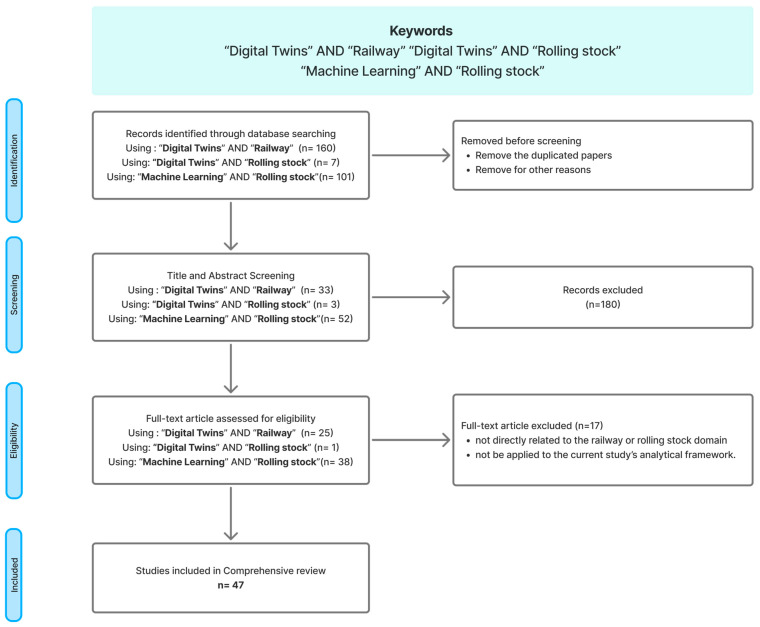
Presents the steps involved in the screening and selection of papers.

The diagram below (Figure 11) presents the procedure for screening and selecting papers.

Following the initial review process described above, a total of 268 studies were found. After applying the review process, which involved analysing the relevance of each study to the research aim, 47 studies were chosen for comprehensive analysis. These consist of 21 studies under the keyword “Digital twins” AND “Railway”, 1 study under “Digital twins” AND “Rolling stock”, and 25 studies under “Machine Learning” AND “Rolling stock”.

## 4. Result

### 4.1. Digital Twins Applications in Railway

#### 4.1.1. Initial Research Insights into Digital Twins for Railway Systems

In this subsection, a selection of notable literature reviews is examined to demonstrate the increasing interest among researchers in the application of digital twins in railway systems. This field of study is still relatively unexplored, although the available literature provides significant insights into ongoing research trends and the developing function of digital twins in the railway sector.

Doubell, et al. [41] Emphasised the increasing trend of digital twins implementation across several industries, driven by the emergence of the Fourth Industrial Revolution. However, the application of digital twins in the railway industry is still limited. The exist-ing studies were reviewed and prospective approaches for using digital twins were suggested in the railway infrastructure data management. These directions include the integration of data from various sources, validation of management paradigms, and the processing of large volumes of data.

Moreover, an additional literature analysis emphasised the growing interest in digital twins [42]; yet, it observed that standardisation in their applications is still lacking. This state-of-the-art literature review employed the PRISMA methodology to investigate the resilience and sustainability of rail and road infrastructure through the implementation of digital twin technologies.

From the reviewed literature, along with the increasing research trend in the field of digital twins, it becomes clear that this area is gaining academic interest. This growing attention has sparked the author’s interest in further examining how digital twins have been utilised within railway systems. In the following subsection, relevant research works will be discussed in detail, categorized into 3 areas: applications related to infrastructure, to rolling stock, and both infrastructures and rolling stock. This categorization is based on the primary focus of each research work, allowing for a clearer understanding of the key areas explored within this domain.

#### 4.1.2. Research on Digital Twins in Railway Infrastructure

From the selected literature, a total of 21 studies were identified that applied digital twins technologies in the context of railway infrastructure. To explain the diverse applications and research directions within this area of study, these papers have been classified into 5 subtopics, each representing a different aspect of digital twins application in railway infrastructure.

##### Data Management Approaches and Framework for Digital Twins of Railway Infrastructure

This subtopic presents studies that focus on the generation of synthetic data to support railway infrastructure applications and the development of digital twins frameworks. Some works have demonstrated how synthetic data can be used to augment limited datasets and improve model training (Table 5).

##### Digital Twins for Defects Visualisation and Enhancing Inspection

This subtopic presents research that explores the application of DTs for defect visualisation and the enhancement of the inspection process in railway infrastructure. The selected studies demonstrate how DTs can support the 3D model of track defects, predict inspection signals, monitor and visualize the operation of level crossings (LC), and simulate fatigue damage. To better understand these applications, each study is discussed, highlighting how digital twins have been utilised across different visualization and inspection contexts (Table 6).

##### Digital Twins for Sustainability

One study demonstrates the potential of digital twins in supporting sustainability objectives within the PCAT concrete slab track system (Table 7).

##### Digital Twins for Condition Monitoring

This subtopic highlights the use of DTs for condition monitoring. The reviewed studies demonstrate how DTs have been employed to monitor various aspects, such as weather conditions and the operational status of railway switches. Additionally, there is a study focused on investigating the causes of deterioration as a foundational step toward the future development of digital twins for condition monitoring (Table 8).

##### Semantic Segmentation for Developing Digital Twin Models

Semantic Segmentation, which involves pixel-wise classification of images or point cloud data to classify into a specific category or class, is considered a promising approach for enhancing the development of DT models (Table 9).

##### Digital Twins for Prediction

This subtopic highlights the use of DTs for prediction (Table 10). The reviewed studies demonstrate how DTs have been applied to forecast rail surface damage and derailment risks, supporting predictive maintenance, enhancing safety, and improving operational efficiency in railway systems.

#### 4.1.3. Research on Digital Twins in Railway Rolling Stock

Following the review of DTs’ application in railway infrastructure, this section focuses on research related to the application of DTs in railway rolling stock (Table 11). Even though the use of digital twins in Train Rolling Stock is not yet widespread, at least there has been research that has started to study the creation of an accurate model of rolling stock, which is a good start to further develop digital twins in the train rolling stock system. Among the several techniques available, laser scanning and vision-based technologies have proven to be successful for capturing detailed structural information.

### 4.2. Overview of Machine Learning Applications in Rolling Stock

After reviewing the research on DTs in infrastructure and rolling stock, it can be seen that the difference in the amount of research on infrastructure and rolling stock is significant. This shows that the use of DTs in rolling stock systems is still in great demand. Therefore, in this section, the research related to the usage of ML in rolling stock systems will be reviewed.

Rolling stock, including critical elements such as wheelsets, bogies, and traction systems, constitutes the foundation of railway operations. These subsystems are essential for ensuring safety, reliability, and performance in contemporary railway networks. Nonetheless, sustaining and enhancing these components present considerable difficulties, particularly because of the complexity of their interconnections and the large amount of data produced during operation.

Machine Learning (ML) is growing as a powerful technology for addressing these difficulties. Utilising advanced algorithms, machine learning facilitates predictive maintenance, real-time monitoring, and defect identification, essential for minimising downtime, enhancing safety, and decreasing operational expenses. The use of machine learning techniques in rolling stock maintenance operations signifies a crucial advancement towards more intelligent and efficient railway systems.

This section provides studies of the Machine Learning techniques used in rolling stock. It classifies the components and their corresponding machine learning methodologies, highlighting the role of these techniques in prediction, detection, classification, and monitoring.

#### 4.2.1. Predicting

Predictive maintenance is essential for reducing breakdowns and enhancing the operational efficiency of rolling stock. Several machine learning techniques have been employed to predict maintenance requirements, save downtime, and improve component reliability. Linear regression and regularisation techniques were employed to predict the maintenance schedule of wheelsets, as noted in [63]. This method effectively forecasts optimal maintenance times through the analysis of previous data, although it is constrained by its susceptibility to outliers and the necessity for data scaling.

Moreover, research utilising moving averages, ARIMA, and seasonal ARIMA (SARIMA) has demonstrated efficiency in developing dependable forecasting models for traction control units (TCU) [64]. These strategies use expert knowledge to analyse onboard diagnostic data and generate predictions. However, issues remain involving data reliance and seasonality, as changes in train performance might reduce forecasting precision. The manual methodology employed in certain models also adds biases, constraining the automation capabilities of machine learning systems.

A related study on Mean Time to Repair (MTTR) [65] employed regression-based techniques, such as linear regression, polynomial regression, and random forests, to forecast downtime and enhance maintenance models. However, challenges remain in terms of data quality, model complexity, and generalisability across railway systems.

Bayesian models, including Bayesian Factorisation and Expectation-Maximization (EM), have been employed in dynamic risk assessment to identify key risk variables in semi-permanent coupler failures [66]. Although these models yield valuable insights using sensitivity analysis, their dependence on high-quality data and complex integrations presents considerable challenges.

Another study introduces a predictive maintenance approach for rolling stock vehicles utilising Machine Learning Algorithms for Remaining Useful Life estimation [67]. The focus is on reducing uncertainty in maintenance schedules, estimating Remaining Useful Life (RUL), and lowering downtime and expenses by effective maintenance scheduling and multi-tier platform execution. Nonetheless, limitations include its dependence on a singular case study, which minimises generalisability, and data reliance, whereby insufficient data affects predictive accuracy. The study underscores the complexity of implementation due to multi-layer architecture and diagnostic integration, insufficient emphasis on specific components important to diverse vehicle types, and restricted validation metrics, which prevent an accurate evaluation of the technique.

The prediction of technical failures in railway door systems utilises models such as Restricted Boltzmann Machines (RBM) and Echo-State Networks (ESN). These approaches effectively predict failures by employing diagnostic data, although they encounter limitations such as data dependence and the static characteristics of training data [68].

Research utilising Random Forests (RF), Quantile Random Forests (QRF), and Principal Component Regression (PCR) has demonstrated considerable advancements in predictive maintenance for calculating the Remaining Useful Life (RUL) of components [69]. Nonetheless, constraints such as insufficient data, complex feature interactions, and challenges in model interpretability remain in limiting wider use.

Additionally, Random Forests (RF) and Missing Value Imputation methods have been employed to predict failure times of railcar wheels and trucks based on wayside detector data [70]. These methods proficiently manage sensor data and enhance railway safety by reducing inspection and maintenance expenses. Nonetheless, constraints such as dependence on a singular dataset, the intricacy of real-world scenarios, and biases from missing data limit the generalisability of these models.

Another research proposes a data-driven methodology for forecasting the service life of rolling stock components through Random Forest Regression (RFR) and Gradient Boosting Regression (GBR) [71]. The primary focus is on forecasting service longevity, addressing the SGR backlog, calculating long-term replacement expenses, and improving asset management approaches. Nonetheless, limitations encompass the emphasis on minor urban and rural networks, potentially limiting generalisability, as well as data restrictions due to reliance on 2020 vehicle inventory statistics. The model’s exclusion of active vehicles could miss significant patterns, while the study underscores the opportunity for model enhancement through the integration of maintenance records and real-time sensor data to improve accuracy and practical relevance.

Within traction converter systems, Long Short-Term Memory (LSTM) networks have exhibited significant precision in forecasting breakdowns and reducing false alarm rates [72]. Although its accuracy, factors such as dataset specificity and temporal reliance on previous data undermine the model’s robustness.

The prediction of energy consumption in metro trains depends on Neural Networks (NN) and data filtration methods to improve energy efficiency and minimise data noise [73]. Nonetheless, factors such as signal noise, limits of closed environments, and data quality persist in affecting the models’ accuracy.

Finally, to improve rail network velocity with a machine learning methodology for predictive maintenance, [74] concentrating on components including bearings, trucks, wheels, and monitoring systems. Methods such as Support Vector Machines (SVM), Decision Trees (DT), distributed learning, and causal analysis were employed to forecast Level 1 (L1) alarms, reduce false alarm rates, and combine various data sources for predictive maintenance. Primary challenges encompass data quality and availability, as model efficacy depends on extensive detection data, and generalizability, where methods may be confined to specific operational situations. Further limitations include the complexity of real-world scenarios, the attainment of a minimal false alarm rate in practice, and the interpretability of sophisticated models such as SVM, which may limit their practical implementation in decision-making.

#### 4.2.2. Detecting

The study examines variations in waveform distortion in railway pantograph observations through the application of Deep Autoencoder [75], clustering techniques, and Principal Component Analysis (PCA). The techniques concentrate on evaluating waveform distortion, recognising patterns, enhancing power quality, and conducting dynamic analysis through the identification of patterns in the recorded waveforms. Significant limitations encompass sample size and diversity, potentially constraining generalisability, as well as challenges with distinguishing the effects attributable to the static versus dynamic observations of pantographs. The complexity of distortion causes and reliance on data quality underscore possible challenges presented by noise or mistakes that could affect model reliability. Moreover, the study’s restricted analytical scope concentrates mainly on waveform distortion.

Fault detection in rolling stock components is essential for maintaining operating safety and reliability. Diverse machine learning methodologies, encompassing Support Vector Machines (SVM), Deep Learning Networks (DNNs), and Wavelet Transform, have been utilised to identify wheel faults, including flat spots, shelling, and non-roundness [76]. Research has shown early detection abilities; nonetheless, difficulties remain in managing class imbalance within datasets and the intricacies of real-world applications.

Methods such as Sparse Autoencoders (SAE), Mahalanobis Distance, and Cluster Analysis have been investigated for detecting out-of-roundness (OOR) in wheels and evaluating the severity and type of damage [77]. Although these technologies exhibit potential, their dependence on simulation-based validation and controlled failure situations could limit their applicability to actual railway systems.

In the realm of fault detection, Convolutional Neural Networks (CNNs), such as Xception and EfficientNet-B7 architectures, have been used for condition monitoring systems [78]. These algorithms combine defect detection with performance evaluation; however, substantial computational resources and environmental considerations present practical deployment challenges.

Hybrid methodologies integrating Multi-Layer Perceptrons (MLP), Support Vector Machines (SVM), together with other techniques such as Decision Trees (DT) and k-Nearest Neighbours (kNN), have been developed for the detection of wheelset failures [79]. Although it has resilience, factors such as sensor requirements, simulation-exclusive data, and limited fault scenarios prevent its practical application.

Detection methods have also been utilised for unsupported sleepers and integrated railway problems employing deep learning methodologies, including Convolutional Neural Networks (CNN), Recurrent Neural Networks (RNN), and Fully Convolutional Networks (FCN) [80]. These investigations underscore their efficacy in identifying faults and assessing severity; however, issues such as a lack of field data, limited sample variety, and the complexity of real-world situations remain unresolved.

The study focuses on wheel flat detection and severity classification using deep learning techniques such as DNN, CNN, and RNN [81]. It aims to improve predictive models and contribute to preventive maintenance by detecting and classifying the severity of wheel flats. However, limitations include the reliance on simulation-based data instead of real-world data and the lack of real-time testing under operational conditions. Additional challenges involve the risk of overfitting, a limited scope focusing solely on wheel flats, and the potential for incomplete feature selection, which may overlook other influencing factors.

Machine learning models, including DNN, CNN, and RNN, have been used to detect and evaluate the severity of railway problems, particularly dipping joints and settlements, by analysing wheel and axle box acceleration (ABA) data [82]. While effective, the study relies on simulation-based data and simplified features, limiting real-world applicability. It underscores the necessity for additional validation and thorough modelling to address the complex interactions of faults.

Utilising machine learning for fault detection enables railway systems to discover problems early, mitigate operating risks, and enhance predictive maintenance plans. Nonetheless, persistent challenges, including data constraints, model intricacy, and generalisability, necessitate additional enhancement to attain viable and scalable solutions.

#### 4.2.3. Classifying

The research provides a comparative examination of Convolutional Neural Networks (CNN), Vision Transformers (ViT), and Compact Convolutional Transformers (CCT) to create an efficient model for the safety inspection of trains, with particular emphasis on the classification of missing bolts in train components [83]. Although the models achieved high accuracy, the study underscores significant limitations, including sample size, as the dataset lacked information on the number of images, consequently impacting generalisability. Environmental variability was insufficiently examined, potentially affecting model performance in real-world scenarios. The necessity for enhanced interpretability was underscored to foster confidence in model predictions during safety inspections. Furthermore, the study’s concentration on missing bolts limits the relevance of its findings to other defect categories or problems.

The research employs t-Distributed Stochastic Neighbour Embedding (t-SNE) to classify the causes of rail contact fatigue (RCF) and to identify clusters of significant loadings associated with prospective damage areas [84]. The analysed key components consist of wheels, axle load, wheel flange, dynamic masses, and wheel trajectories. The experimental analysis offers interesting insights; nonetheless, the study is limited by its singular crossing focus, measurement limitations, and insufficient model validation. The investigation primarily focuses on high-impact load conditions, possibly ignoring other contributing aspects.

#### 4.2.4. Monitoring

An intelligent IoT sensing system for rail vehicle running states based on artificial neural networks (ANN), sigmoid activation functions, model training and conversion, and offset time window methods is proposed [85]. The system enables real-time detection of the complex operational status, offering advantages including low energy consumption, cost efficiency, data collection and processing, deployment, and visualisation. However, there are significant challenges and limitations to acknowledge. Data dependency influences the model’s accuracy, as it is significantly contingent upon the quality and quantity of the training data. Sensor limitations occur because the study primarily concentrated on acceleration data, overlooking other potentially important inputs that could enhance accuracy and robustness. Generalisability is a concern, as findings may not be universally applicable across many contexts. The limits of real-time processing indicate that processing speed and efficiency may vary based on model complexity and the capabilities of IoT microcontrollers.

Another research [86] aims to improve the accuracy of 3D point clouds using supervised machine learning for the automated inspection of train couplers. Algorithms including Linear Regression Models, Regression Trees, Gaussian Regression Models, Support Vector Machines (SVM), and Ensembles of Trees enhance target localisation precision in dynamic and unstructured situations. Challenges encompass the environmental variables that influence sensor data accuracy, limits of depth sensors, including inadequate precision and resolution, and the necessity for a more extensive and diverse dataset to enhance model resilience. Moreover, reliance on supervised learning presents challenges, as collecting labelled training data can be expensive and labour-intensive, particularly in complex situations.

Finally, the fretting fatigue damage in solid railway axles was evaluated by unsupervised machine learning and non-destructive testing methodologies [87]. An Unsupervised Intelligent Classification System utilising Artificial Neural Networks (ANN) efficiently categorised acoustic emission (AE) data into classifications, including fretting damage and background noise, alongside the application of a k-SOM clustering approach. Significant limitations encompass the utilisation of artificial notches, which may fail to mimic real-world conditions, the testing of a single specimen that impacts generalisability, and the lack of previous studies on structural health monitoring (SHM) for axles. The study emphasises the efficacy of AE and ANN while recommending the investigation of alternative machine learning models and hybrid methodologies. Table 12 presents a comprehensive overview of machine learning (ML) techniques applied to various components of rolling stock across different purposes, including Predicting, Detecting, Classifying, and Monitoring.

## 5. Discussion

The applications of DTs in railway systems and machine learning in rolling stock have been explored from multiple perspectives. This section aims to analyse how these technologies could enhance strategies for reducing aluminium and steel waste, which were identified as the highest portion of the material in high-speed train rolling stock waste, particularly in the context of the circular economy and the 10R principles.

### 5.1. Digital Twins in Railway Systems

The review of 22 research studies on the application of digital Twins technology in railway systems revealed a strong focus on infrastructure management, with 21 of the studies primarily targeting this area. In contrast, only one study was dedicated to rolling stock, indicating a relatively limited application of DTs in this domain. While most studies concentrate on data management and using DTs for improving the system, relatively few address the integration of the circular economy and 10R principles. This imbalance highlights a critical research gap, suggesting the need for further development in applying DTs to support sustainability goals.

### 5.2. Applications of Machine Learning in Rolling Stock

After reviewing 25 research studies, the applications of Machine Learning in rolling stock can be classified into four primary categories: prediction, detection, classification, and monitoring. Furthermore, insights obtained from Table 5 reveal that these applications have been executed across multiple rolling stock components.

**Figure 12:**
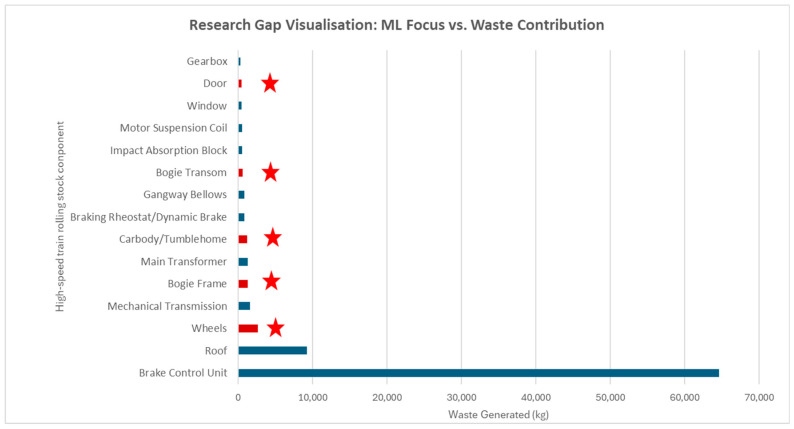
Top 15 rolling stock components ranked by waste generated, ★ indicates components where Machine Learning applications have been identified in existing studies.

A comparative analysis between the 15 rolling stock components that generate the highest volume of waste, as indicated in Table 3, and the existing applications of Machine Learning (ML) in rolling stock research, reveals a significant research gap. Of the top 15 waste-generating components, only four have been examined in previous studies employing machine learning approaches, as shown in Figure 12, namely in the domains of prediction, detection, and classification. Significantly, none of these studies have concentrated on monitoring, a fundamental function of digital twins essential for enabling monitoring of component degradation and enhancing circular economy and 10R principles to improve sustainability and minimize material waste in high-speed train rolling stock.

**Figure 13:**
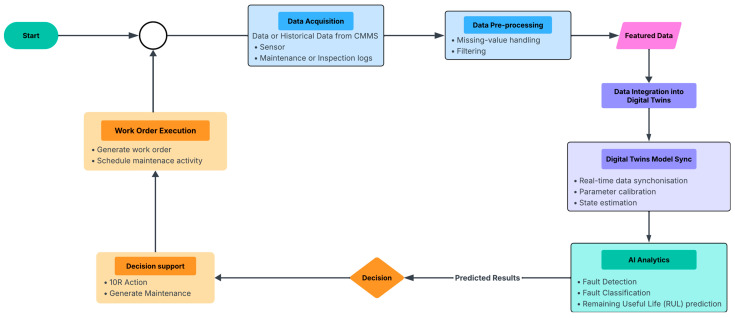
Automated Workflow for Condition Monitoring and 10R Integration.

Based on the reviewed studies, it is evident that no current applications of condition monitoring in focused components. To address this limitation, an automated workflow is proposed in this study. This framework (Figure 13) highlights the incorporation of circular economy and 10R principles in component management as a pathway to sustainable resource utilisation. By systematically applying these principles, the framework provides guidance for optimising material flows, reducing unnecessary losses, and advancing railway systems towards a more circular and resilient model.

### 5.3. Quantitative Indicators for Evaluating Circular Economy Performance

To establish a measurable foundation for future research, several quantitative indicators are proposed to assess both the economic and environmental performance of rolling stock systems under Circular Economy (CE) principles. These indicators—Net Present Value (NPV), Embodied Carbon, ISO-based Life Cycle Cost (LCC), and Carbon Accounting—serve as key tools for evaluating sustainability outcomes. Economic indicators such as NPV can be used to assess the long-term financial viability of asset management strategies:

$$NPV=\sum_{t=1}^{n}\frac{R_{t}-C_{t}}{(1+r{)}^{t}}$$

where $R_{t}$ and $C_{t}$ represent the revenue and cost at time t, and r is the discount rate. This formulation helps identify cost-efficient maintenance or refurbishment strategies over the asset lifecycle.

From the environmental perspective, Embodied Carbon is a crucial indicator that quantifies the greenhouse gas (GHG) emissions associated with material production, use, and end-of-life processes:

$$EC=\sum_{i=1}^{n}\left(m_{i}\times EF_{i}\right)$$

where $m_{i}$ is the mass of material i and $EF_{i}$ is its emission factor. Integrating these indicators with ISO-based LCC and Carbon Accounting frameworks will provide a more comprehensive understanding of both cost and environmental trade-offs, forming a quantitative basis for evaluating CE-oriented digital twin applications in future studies.

### 5.4. Potential Challenges of Digital Twins and AI Applications in High-Speed Train Rolling Stock

The integration of digital twins and AI to enhance the implementation of the circular economy in high-speed train rolling stock represents a novel and promising research direction. Nevertheless, several challenges associated with the practical adoption of digital twins and AI remain. These include issues related to data integration, data privacy, and the difficulties of applying AI models to real-world scenarios. As discussed in the following sections.

#### 5.4.1. Data Integration and Interoperability

One of the major challenges in enhancing the circular economy using digital twins and AI for high-speed train rolling stock is the integration of heterogeneous data from multiple sources, including operators and maintenance providers, and multiple components, leading to difficulties in achieving real-time interoperability.

#### 5.4.2. Data Privacy and Governance

Data privacy is another issue, particularly when digital twins involve real-time monitoring. Sensitive operational data, such as component health or maintenance schedules, may raise privacy concerns. Establishing clear data ownership, access control policies, and a secure data-sharing mechanism is essential.

#### 5.4.3. AI Robustness, Bias, and Interpretability Challenges

AI has been successfully and widely adopted across various domains. However, applying AI models to real-world situations still faces several limitations and challenges, both internal and external. The examples of the internal challenges are a biased training dataset or an imbalanced dataset. A biased training dataset or an imbalanced dataset may lead to inaccurate fault detection or misleading sustainability assessments. One prominent issue is the zero-fault dataset. To address this limitation, Chen, et al. [88] proposed a Relation Network with Out-of-Distribution Data Augmentation (OOD-RN) combined with a soft Brownian generator, enabling effective learning and improving the model’s capability to detect faults under zero-fault conditions. Since many components of high-speed train rolling stock operate under rotation motion, the Shrinkage Mamba Relation Network (SMRN) has been introduced to facilitate fault detection in rotation machinery under zero-fault conditions [89]. External factors, on the other hand, involve challenges such as adversarial data. The adversarial data can lead to inaccurate decisions with substantial consequences. Tasneem and Islam [90] addressed this issue by developing an improved adversarial training approach that leverages explainable AI-guided feature and data augmentation techniques to strengthen the AI model.

### 5.5. Broader Implementation Challenges

Beyond the technical challenges discussed in the previous sections, several broader factors can influence the practical implementation of digital twins and AI applications in high-speed train rolling stock. These include external or regulatory considerations, model validation constraints, and workforce adaptation. While these aspects are not explored in depth within this state-of-the-art review, acknowledging them is essential for understanding the real-world readiness and sustainability of digital twins and AI-based frameworks.

## 6. Conclusions

This state-of-the-art review has emphasised the transformative potential of new technologies, specifically digital twins and machine learning, in enhancing the circular economy in high-speed train rolling stock. In the field of infrastructure, digital twins have shown effectiveness in improving predictive maintenance and real-time monitoring, thereby dramatically decreasing operational interruptions and maintenance expenses. These characteristics highlight their significance as essential instruments for enhancing resource allocation and augmenting system resilience.

The limited adoption of digital twins in rolling stock indicates a distinct possibility for additional research. Although digital twins applications in infrastructure are extensively documented, the domain of rolling stock is comparatively underexplored, with a limited number of studies addressing this topic. Closing this gap could facilitate enhanced rolling stock performance via sophisticated data integration and real-time monitoring methods.

Machine Learning provides further opportunities for enhancing the prediction, detection, and classification of rolling stock components’ conditions, and especially, increasing monitoring capabilities. This highlights a critical research gap and the need for future studies to explore ML-based monitoring strategies for high-waste components and the integration of machine learning with digital twins to enable real-time system performance monitoring, interdisciplinary data exchange, and sustainable lifecycle management in accordance with circular economy and 10R principles.

## Figures and Tables

**Table 1:** Examples of the implementation of the circular economy in the railway industry.

Context/Purpose	Focus Area	CE Target	Reference
- Evaluated end-of-life rolling stock with a focus on asset recycling and energy recovery, calculating recyclability and recoverability rates	Rolling stock	Recycle /Recovery	Kaewunruen, et al. [10]
- Developing a theoretical framework for the implementation of circular economy in the ballasted track system.	Infrastructure	Overall	Koohmishi, et al. [17]
- Examined reused ballast with tire-derived aggregates, showing performance improvements and alignment with circular economy principles.	Infrastructure	Recycle /Reuse	Lenart and Karumanchi [18]
- Applied foamed polyurethane to mitigate excessive subgrade settlement, proposing empirical formulas to describe its expansion mechanism.	Infrastructure	Refurbish /Repair	Huang, et al. [19]
- Using the recycled rubber elements in track substructure to increase confinement sub-ballast and shoulder ballast in a hybrid high-speed rail track	Infrastructure	Recycle	Indraratna, et al. [20]
- Developed and validated numerical models of small-scale brick-lined metro tunnels to identify critical locations for design, maintenance, life extension, and end-of-service-life stages	Infrastructure	Reuse /Recycle	Chen, et al. [21]

**Table 2:** Summary of total weight of rolling stocks (kg), recyclability rate (%), and recoverability rate (%) comparing with three types of rolling stocks.

Type of Rolling Stock	Results					
	Total Weight (kg)	Total Waste (kg)	Fraction of Total Waste and Total Weight	Recyclability Rate	Energy Recovery Rate	Recoverability Rate
Freight train	8,000,000	520,192	6.5%	92.8%	0.9%	93.7%
Passenger train	168,373.5	15,661.9	9.3%	89.2%	89.2%	91%
High-speed Train	265,000	88,201.1	33.3%	61.4%	61.4%	73.9%

**Table 3:** Top 15 components ranked by waste generated (in kilograms).

Component of Rolling Stock	Type of Material	Weight (kg)	Waste (kg)	Percentage %
Brake Control Unit	Aluminium/Steel/Composites	97,944.00	64,643.00	74.56
Roof	Aluminium/Steel	14,071.50	9287.20	10.71
Wheels	Steel R7	44,069.50	2644.20	3.05
Mechanical Transmission	Aluminium Alloys/Steel	2438.00	1609.10	1.86
Bogie Frame	Steel plate/Cast steel/Composites	22,048.00	1322.90	1.53
Main Transformer	Steel/Aluminium	1961.00	1294.30	1.49
Car body/Tumblehome	Aluminium/Steel/Composites	20,749.50	1245.00	1.44
Braking Rheostat/Dynamic Brake	Aluminium/Steel	1139.50	866.00	1.00
Gangway Bellows	Silicon-coated fabric	8559.50	856.00	0.99
Bogie Transom	Steel plate/Cast steel/Composites	9805.00	588.30	0.68
Impact Absorption Block	Aluminium	5644.50	564.50	0.65
Motor Suspension Coil	Steel	8559.50	513.60	0.59
Window	Glass	4902.50	490.30	0.57
Door	Aluminium/Steel	7340.50	440.00	0.51
Gearbox	Steel	5512.00	330.70	0.38
Total Waste (kg)	86,695.10			

**Table 5:** Studies on Synthetic Data Generation and Digital Twin Frameworks for Railway Infrastructure.

Reference	Purpose	Method/Approach	Key Findings
Salierno, et al. [43]	- Developing a digital twins architecture for switch points - Producing synthetic data and comparing the resultant patterns with raw data	- Using several models - Evaluating the fidelity of synthetic data patterns using FID, precision, recall, and coverage scores	- TimeGAN demonstrated outstanding performance
Hu, et al. [44]	- Enlarging the range, type, and number of discriminative crack features for improving hybrid algorithm performance with uncertain inspection data	- Using digital twins to generate synthetic data	- Enhancing the performance of the hybrid algorithm when dealing with uncertain inspection data
Adeagbo, et al. [45]	- Establishing the architectural foundation for digital twins in railway infrastructure, enabling data integration, simulation, and real-time monitoring	- Developing a conceptual framework for Structural Health Monitoring in advanced rail transit systems to support the transition from digital shadows to digital twins	- Highlighting the potential of digital twins to revolutionize Advanced rail transit systems maintenance and safety with predictive and integrated monitoring, while noting technical and organizational challenges.
Vieira, et al. [46]	- Proposing a conceptual framework for the digital twinning of engineering physical assets	- Defining 9 dimensions: hierarchy, connection, synchronisation, geometric representation, non-geometric representation, intelligence, interface, accessibility, and autonomy - Implementing the framework in road and rail infrastructure projects in Portugal	- Advocating a nuanced and flexible understanding of digital twins through harmonized assessment approaches (LoDT and UNI-TWIN)
Barari [47]	- Utilising live digital twins to determine optimal sensor placements in the LRT system.	- Applying a four-stage framework: developing a high-fidelity (HF) model using multi-physics simulations, calibrating a low-fidelity (LF) model for fault scenarios, validating LF accuracy against physical responses, and optimising sensor placement to maximise fault detection while reducing data load.	- LIVE DT methodology provides a flexible, cost-effective framework for implementing smart diagnostic and prognostic systems in complex assets
Barari [48]	- To develop a dynamic and adaptive virtual model of a physical asset that supports proactive maintenance, diagnostics, and prognostics - To enable real-time monitoring and intelligent decision-making for structural systems	- Combining physics-based models with data-driven analytics to predict remaining useful life (RUL), identify potential issues proactively, and forecast future system behavior based on real-time sensor data	- Digital twin can facilitate smart inspection, predictive maintenance, and failure prediction by employing multi-physics simulations across different phases of asset life cycle
Kochan, et al. [49]	- Virtual prototyping and simulation of operational scenarios	- Mathematical representation of railway infrastructure using graph theory	- Formal multigraph-based model (Multigraph IS) provides an effective and flexible way to represent the physical railway infrastructure in a digital environment

**Table 6:** Applications of Digital Twins in Railway Defect Visualisation and Inspection.

Reference	Context/Purpose	Method/Approach	Key Findings
Ahmad, et al. [50]	- Developing a 3D model to analyse defects occurring in railway trackst	- Modelling a rail according to the RF14 profile and using spline-based technology for complete track - Integrating defects into model using Reflectors A.10 and B.5	- Producing a digital twins model that is adaptable and appropriate for broader implementation
Djordjević, et al. [51]	- Developing a digital twins concept to monitor level crossing (LC) operations and identify potential faults and failures	- Employing sensors and data acquisition techniques to monitor the state of level crossings (LCs)	- Improving system reliability and operational safety
Hamarat, et al. [52]	- Exploring the use of digital twins to support real-time data integration, simulate fatigue damage, and validate models for cost-effective railway maintenance	- Employing a combination of peridynamic theory, cyclic loading simulations, and numerical implementations	- The non-local nature of peridynamics allows for a more accurate representation of complex crack behaviours compared to traditional methods
Ramatlo, et al. [53]	- Utilising a hybrid physics-based and data-driven model (VAE) to predict inspection signals, demonstrating accurate predictions under various settings	- Modelling wave propagation response using 3D FE and 2D semi-analytical FE, and generating response signals from a physics-based model to train a data-driven model based on a VAE	- Effectively predicting inspection signals using the trained VAE

**Table 7:** Applications of Digital Twins for Sustainability.

Reference	Context/Purpose	Method/Approach	Key Findings
Borjigin, et al. [54]	- Demonstrating the potential of digital twins in supporting sustainability objectives within the PCAT concrete slab track system	- Employing digital twins to evaluate the bill of quantities (BOQ) of materials	- Identifying that components such as rubber pads, bituminous layers, and grout had considerable environmental impacts and were the most expensive materials

**Table 8:** Applications of Digital Twins for condition monitoring.

Reference	Context/Purpose	Method/Approach	Key Findings
Kampczyk and Dybel [55]	- Employing digital twins to facilitate the monitoring of the weather surrounding railway turnouts	- Using the T_szyn_ WS1 WIFI measuring station integrating multiple sensors with real-time wireless transfer and calculating the second temperature difference indicator and average temperature as diagnostic indicators for turnout health.	- Providing valuable diagnostic insights (e.g., rail buckling, thermal stresses) through the second temperature difference indicator - Supporting automated data acquisition, personalised measurement configuration, and integration into digital twins frameworks.
Boschert and Rosen [56]	- Introducing the concept of digital twins focusing on physical and virtual model integration to improve railway switch monitoring systems	- Combining physics-based simulation with sensor data-driven analysis	- Better maintenance strategies through synchronized data
Ekberg, et al. [57]	- Investigating rail and wheel health, aiming to detect, monitor, and predict the evolution of important health parameters for the creation of digital twins	- Analysing deterioration causes from plastic deformation and wear, mechanical deterioration due to fatigue cracking, and other deterioration forms	- Integrating physical modelling, data analysis, and advanced inspection methods enhances the prediction and management of wheel and rail deterioration, especially RCF.
Avsievich, et al. [58]	- Enhancing the diagnostics and monitoring of railway infrastructure by analysing the dynamic interaction between rolling stock and track elements	- Employing the FE to assess the stress-strain state of the track, integrates advanced sensor technology and IoT to enable real-time data collection	- The use of high-precision accelerometers, combined with a finite element 3D model, enables accurate stress-strain analysis of railway tracks under operational loads.

**Table 9:** Applications of Semantic Segmentation in Enhancing Digital Twin Models.

Reference	Context/Purpose	Method/Approach	Key Findings
Ton, et al. [59]	- Developing a digital twins model of the catenary arches in the Dutch railway system using semantic segmentation	- Using a mobile laser scanner to capture the geometric structure - Applying semantic segmentation to categorise parts of the structure - Employing PointNet++, SuperPointGraph, and Point Transformerfor gor point cloud data segmentation	- The modified PointNet++ achieved the highest mean class Intersection over Union (IoU) of 71% - Deep learning approaches are effective for automating the semantic segmentation of railway infrastructure point clouds

**Table 10:** Applications of digital twins for prediction.

Reference	Context/Purpose	Method/Approach	Key Findings
Ahmad, et al. [60]	- Developing digital twins to predict rail surface damage due to RCF in heavy haul railway operations	- Using advanced simulations and multibody simulation techniques to estimate contact geometry and wear patterns, combined with model-based system engineering to enable interaction between digital twins and the physical railway system	- Digital twins can enhance predictive maintenance and operational efficiency in heavy haul railways
Bernal, et al. [61]	- Improving derailment risk predictions within railway systems by developing an augmented digital twin	- Integrated machine learning techniques with multibody dynamic simulations; used a surrogate model trained on extensive simulation data to enable ATO systems to make informed control decisions	- Enhancing safety and operational efficiency, reduces wear and rolling contact fatigue, and supports sustainability and cost-effectiveness

**Table 11:** Applications of digital twins in railway rolling stock.

Reference	Context/Purpose	Method/Approach	Key Findings
Avizzano, et al. [62]	- Developing a robust algorithm for image stitching and reconstruction of the exterior surfaces of rolling stock	- Proposing a Novel Kalman Filter with a Multiple-Hypothesis Measurement Model; applied laser scanning and vision-based technologies to capture detailed structural information	- Successful capturing structural data of rolling stock include laser scanning and vision-based methods.

**Table 12:** Summary of the best Machine Learning Techniques for Rolling Stock Components.

Purpose	Reference	Component	Best ML Techniques
Predicting	Kulikov, et al. [63]	Wheelsets	Linear regression
	Ferdous, et al. [64]	Traction Control Unit (TCU)	ARIMA and SARIMA
	Ragala, et al. [65]	Not specify components	Linear and polynomial regression
	Appoh and Yunusa-Kaltungo [66]	semi-permanent coupler	Bayesian methods
	Nappi, et al. [67]	Several components	The paper does not detail specific ML techniques used.
	Fink, et al. [68]	railway door system	Combined approach of RBM and ESN
	Li and He [69]	wheels, Bogies	Random Forest (RF) and Quantile Random Forest (QRF)
	Li and He [70]	Wheel, Bogies	Random Forests (RF)
	Mistry and Hough [71]	service life of various components of rolling stock	Gradient Boosting Regression (GBR)
	De Simone, et al. [72]	Traction Converter Cooling system	Long Short-Term Memory (LSTM)
	Fernández, et al. [73]	several key components of rolling stock	Neural Networks (NN)
	Li, et al. [74]	Bearings, Bogies, Wheels, Alarms and Monitoring systems, and Overall Equipment Health	Support Vector Machines (SVM)
Detecting	Salles, et al. [75]	Pantograph, Locomotive	DAE
	Krummenacher, et al. [76]	Wheels	Support Vector Machines (SVM) and Deep Neural Networks (DNN)
	Magalhaes, et al. [77]	Wheels	Sparse Autoencoders and Mahalanobis distance
	Chung and Lin [78]	Wheels	EfficientNet-B7
	Shaikh, et al. [79]	Wheelset	MLP-RF
	Sresakoolchai and Kaewunruen [80]	Car body, Bogies, Wheelsets, Primary Suspension, and Secondary Suspension	CNN
	Sresakoolchai and Kaewunruen [81]	Axle Box Accelerations, Wheels, Rails, Vehicle weight and speed and sleeper spacing	DNN
	Sresakoolchai and Kaewunruen [82]	Wheel, Axle box Acceleration (ABA)	CNN
Classifying	Alif, et al. [83]	Bolts	Vision Transformer (ViT)
	Sysyn, et al. [84]	Wheels, Axle load, Wheel Flange, Dynamic Masses, Wheel Trajectories	t-distributed stochastic neighbor embedding (t-SNE)
Monitoring	Zhou, et al. [85]	Rail Vehicle	ANN
	Vithanage, et al. [86]	automatic train coupler	EGPR and SWLR
	Carboni and Zamorano [87]	Axles	ANN

## Data Availability

All data supporting the findings of this study are available within the article itself and through the referenced sources.
